# Comparison of the clinical efficacy of rhythmic transcranial magnetic stimulation protocols in patients with depression

**DOI:** 10.1192/j.eurpsy.2025.2126

**Published:** 2025-08-26

**Authors:** A. N. Pomytkin, A. Y. Komarova

**Affiliations:** 1 Laboratory of neuroimaging and multimodal analysis, Mental Health Research Center, Moscow, Russian Federation

## Abstract

**Introduction:**

Transcranial magnetic stimulation (TMS) is a method of non-invasive stimulation of the cerebral cortex with an alternating magnetic field, used in the therapy of patients with depression, including cases where drug therapy is ineffective. The currently widely used high-frequency stimulation protocols with the application of an inductor to the projection of the left dorsolateral prefrontal cortex (LDLPC) have confirmed their effectiveness, while protocols with a significantly shorter procedure time are appearing in clinical practice. In this study, a clinical comparison of two protocols was conducted.

**Objectives:**

To conduct a comparative assessment of the clinical effect of TMS using theta-burst stimulation and high-frequency (10 Hz) stimulation.

**Methods:**

The study included 34 patients (mean age 27.2±2.1 years) with a diagnosis F31-F34 and clinical depression confirmed by psychometric assessment data (HDRS=21.7±5.6). Patients received stimulation in the projection of the left dorsolateral prefrontal cortex for 3 weeks: 17 patients (group 1) received high-frequency stimulation (10 Hz) and 17 patients (group 2) intermittent theta burst stimulation. Symptom reduction, according to psychometric assessment, corresponding to 50% or more, was chosen as the criterion for response to treatment.

**Results:**

By the end of the study, the number of patients in the total sample who responded to treatment corresponded to 55.9% (n=19). Comparison of the effectiveness of the protocols showed that the group statistically significantly differed in the number of responders (p<0.05; 0.01): in group 1 47.06% (n=8), in group 2 64.71% (n=11). Patients who received theta-burst stimulation had a faster rate of symptom reduction and demonstrated higher adherence to treatment.

**Conclusions:**

Thus, a comparative study of the effectiveness of two TMS protocols demonstrated a higher effectiveness of theta-burst stimulation, however, it is worth noting the overall effectiveness of this method regardless of the stimulation parameters, as well as a fairly rapid development of the clinical effect.

The study was supported by RSF grant 22-15-00437.

**Disclosure of Interest:**

None Declared

